# Appointment waiting times and education level influence the quality of bowel preparation in adult patients undergoing colonoscopy

**DOI:** 10.1186/1471-230X-11-86

**Published:** 2011-07-28

**Authors:** Wah-Kheong Chan, Arjunan Saravanan, Jeeta Manikam, Khean-Lee Goh, Sanjiv Mahadeva

**Affiliations:** 1 Division of Gastroenterology and Hepatology, Department of Medicine, Faculty of Medicine, University of Malaya, Kuala Lumpur, Malaysia

## Abstract

**Background:**

Risk factors for poor bowel preparation are recognized to be independent of the type of bowel preparation method used. Patient and administrative factors influencing bowel preparation are known to vary in different healthcare systems.

**Methods:**

A prospective, cross-sectional study of patients undergoing colonoscopy in an Asian tertiary centre was conducted to identify risk factors associated with poor bowel preparation, and to evaluate the impact of poor bowel preparation on technical performance and patient comfort.

**Results:**

Data on 501 patients (mean age 60.1 ± 14.0 years old, 51.2% males, 60.9% with secondary education or higher) was available for analysis. Poor bowel preparation was present in 151 patients (30.1%). Lower education level (OR = 2.35, 95% CI = 1.54 - 3.60), colonoscopy appointment waiting time beyond 16 weeks (OR = 1.86, 95% CI = 1.04 - 3.37) and non-adherence to bowel preparation instructions (OR = 4.76, 95% CI = 3.00 - 7.55) were identified as independent risk factors for poor bowel preparation. Poor bowel preparation was associated with a lower cecal intubation rate (78.1% versus 98.3%, p < 0.001), prolonged total colonoscopy time (25.4 ± 12.6 minutes versus 16.7 ± 10.2 minutes, p < 0.001), and increased patient discomfort during colonoscopy (patient with moderate to severe abdominal discomfort 31.8% versus 3.2%, p < 0.001).

**Conclusions:**

Education levels and appointment waiting times, in addition to non-adherence to bowel preparation instructions, increase the risk of poor bowel preparation in adult patients undergoing colonoscopy. The latter has a significant impact on colonoscopy performance and patient comfort.

## Background

The incidence of colorectal cancer is rapidly increasing in the Asia-Pacific region [1]. Colonoscopy remains the most accurate tool in diagnosing this condition and is now advocated in many regions to be the modality of choice for screening and surveillance [2]. Apart from visual diagnostic capabilities, it facilitates tissue sampling for histological confirmation and offers therapeutic potential in the form of polyp/early cancer resection [3].

However, the diagnostic accuracy of colonoscopy remains dependent on the quality of bowel preparation. Poor bowel preparation has been shown to significantly impede the diagnostic ability of standard colonoscopy. Previous studies have reported that detection of neoplastic lesions was significantly reduced in patients with poor bowel preparation [4]. While some studies [5] reported that inadequate bowel preparation reduced the detection of small colonic lesion (polyps ≤ 9 mm) others [6] observed a similar trend regardless of the size of colonic lesions.

Apart from a lower diagnostic yield, poor bowel preparation has additionally been associated with incomplete colonoscopy examinations, prolonged procedural duration, and increased procedural difficulty. In a European multi-centre trial, it was demonstrated that high-quality bowel preparation was associated with a higher rate of complete examination, shortened procedural time and lower rate of procedure difficulty when compared with low-quality bowel preparation [6]. Additionally, an inadequate bowel preparation has been estimated to result in an estimated 12% - 22% increase in overall colonoscopy cost compared to good-quality bowel preparation [7].

With these factors in mind, it is imperative that colonoscopy is performed with a high-quality bowel preparation to obtain the best yield. Hence, evidence-based bowel preparation methods have evolved over time with emphasis on efficacy as well as safety, tolerability and affordability [8]. However, despite advances in bowel preparation methods, bowel preparation has remained poor in reported series of patients undergoing colonoscopy. While some studies [6] have reported an inadequate bowel preparation rate of 10%, the rate is often higher and over 20% in other studies [5,9,10].

Risk factors for poor bowel preparation have been recognized to be independent of the type of bowel purgatives used. A range of patient and administrative-related factors such as colonoscopy starting time, failure to follow preparation instructions, inpatient status, procedural indication, specific drugs, male gender and various co-morbidities have been found to be associated with poor bowel preparation in several studies conducted in mostly Western patients [6,9]. In a study from Korea [10], older age, a history of diabetes mellitus and past surgery were identified as risk factors for poor bowel preparation. As demographics and clinical practice are known to vary between different health-care systems, particularly in less developed countries, factors influencing quality of bowel preparation in Asians remain uncertain. The purpose of our study was to identify risk factors associated with poor bowel preparation in Malaysian patients undergoing colonoscopy and to evaluate the impact of poor bowel preparation on technical performance and patient comfort.

## Methods

### Patients and study design

This study was conducted in a large tertiary endoscopy unit, using a prospective, cross-sectional design. The unit has an open-access policy for colonoscopy referrals, i.e. patients were referred directly from both primary care and specialist (secondary care) clinics. Consecutive adult patients undergoing colonoscopy between October 2006 and March 2007 for various indications were enrolled. Exclusion criteria included cases with an incomplete colon examination not related to bowel preparation, e.g. obstructing tumor and acute lower gastrointestinal bleeding not amenable to a standard bowel preparation. Written informed consent was obtained from all patients and the study was approved by the ethical committee of this institution.

Patients were interviewed by a single investigator (AS) on the day of their scheduled colonoscopy appointment and a self-designed questionnaire was used for data collection. Information acquired included basic demographic data, body mass index (BMI), in-patient or out-patient status, timing of colonoscopy i.e. morning or evening, waiting time for colonoscopy appointment (from patient receiving the appointment to the day the procedure was performed), indication for colonoscopy, history of previous colonoscopy, concomitant medical illness, past surgery and compliance to bowel preparation. This information was collected prior to the procedure. Additional data relating to technical aspects of colonoscopy, quality of bowel preparation and patients level of discomfort were collected after the procedure. All patients received a combination of Midazolam 2.5 mg to 5 mg and Pethidine 25 mg to 50 mg as sedation prior to colonoscopy. The dosage of these medications was based on patient's age and concomitant medical illness. Colonoscopy was performed using standard video-endoscopes with variable stiffness (CF 160AL, Olympus, Tokyo, Japan). Various grades of endoscopist were involved including consultants, specialists/registrars and trainees under supervision. The endoscopists were categorized as senior if they had performed 200 or more colonoscopies independently and trainees if they had performed less than 200 colonoscopies independently.

Bowel preparation was graded by endoscopists who were blinded to data on patients' compliance. The grading scale of bowel preparation that endoscopists reported independently to the investigator is described below. Technical aspects of colonoscopy that were collected for analysis included the following: i) cecal intubation time i.e. time taken to reach the cecum after colonoscope insertion through the anus, ii) total colonoscopy time i.e. time from colonoscope insertion till withdrawal from the anus, iii) total amount of fluid used for flushing, and iv) adenoma detection (all adenomas were of a minimum size of 0.5 cm and were subsequently confirmed by histology). Cecal intubation and total colonoscopy times were measured using a mobile phone stopwatch. No adjustment was made for time spent to perform therapeutic work.

Following completion of colonoscopy and recovery from sedation, all patients were interviewed by the same investigator, who was blinded to the quality of bowel preparation during the procedure. Patients' level of comfort during and 1-hour post colonoscopy was assessed using a 4-point Likert scale (1, no discomfort; 2, mild discomfort; 3, moderate discomfort; 4, severe discomfort/abdominal pain).

### Bowel preparation and grading

A standardized bowel preparation regime consisting of bisacodyl and low-residue diet followed by a 2-liter polyethylene glycol and electrolyte lavage solution (PEG-ELS) is used for all patients undergoing colonoscopy at this institution. This regime has previously been shown to be as effective but better tolerated than a 4L PEG-ELS preparation [11-13]. Patients will take bisacodyl 10 mg on the first and second nights and will be on a low-residue diet on the second and third days of bowel preparation. On the third day, patients will take the PEG-ELS within 1 hour from 1800 hour till 1900 hour in preparation for colonoscopy on the following morning. Patients are allowed only plain water after starting intake of PEG-ELS till colonoscopy. For patients whose colonoscopy appointment is scheduled in the afternoon, PEG-ELS will be taken within 1 hour from 0800 hour till 0900 hour on the third day which is the colonoscopy day.

Non-compliance to bowel preparation was defined as the patient's admitted failure to follow prescribed instructions on bowel preparation including volume of bowel preparation solution to be taken, duration within which the bowel preparation solution should be completed and adherence to dietary restrictions. Standardized bowel preparation instructions were given verbally by the Endoscopy Unit receptionist who was trained to provide these instructions as a daily routine. Patients and relatives (in cases where patients were unable to read for whatever reason) were given a brochure in both English and Malay languages as a reminder of bowel preparation instructions. The verbal instructions and brochure were given at the time of booking for the colonoscopy appointment.

The quality of bowel preparation in our study was assessed by the endoscopist independently and categorized as excellent (adequate visualization of the entire colon without flushing and suction), good (adequate visualization of the entire colon (> 90%) with clear fluids requiring minimal suction and no or very minimal flushing), fair (unsatisfactory visualization of all or part of the colon with colored fluid and liquid feces that need suction and flushing) and poor (unsatisfactory visualization of all or part of the colon with colored fluid and feces that need suction and flushing and re-examination need to be considered), based on previously described bowel preparation scale [14].

A pilot study was carried out to address the inter-observer variability of the bowel preparation grades described above. Three independent consultant gastroenterologists from this institution graded the quality of bowel preparation of thirty patients and the result was analyzed using kappa statistics. For the categories described above, it was observed that kappa values for inter-observer rates were as follows: excellent κ = 0.74, good κ = 0.24, fair κ = 0.34 and poor κ = 0.70. As a result, although the grading preparations were recorded as above, they were re-categorized for the purposes of subsequent analysis as follows: good (i.e. excellent), intermediate (combination of good & fair) and poor (as above).

### Statistics

Data were analyzed using a standard statistical software program (SPSS 11.5). Several continuous variables were dichotomized for univariate analysis including age, BMI and waiting time for colonoscopy appointment. Categorical variables were analyzed using chi-square test. Continuous variables were expressed as means with standard deviations and analyzed with either student's t-test (parametric) or Mann-Whitney test (non-parametric) where appropriate. The quality of bowel preparation was dichotomized to poor and non-poor (i.e. good & intermediate combined) and independent risk factors associated with poor bowel preparation were identified using logistic regression analysis. Significance was assumed at a p-value of < 0.05.

## Results

**Figure 1:**
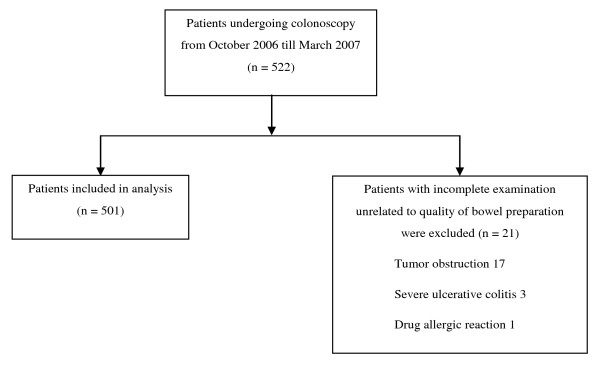
**Study flow diagram**.

Of the 522 consecutive patients who attended for colonoscopy during the study period, 501 patients were eligible. Twenty one patients with incomplete examination unrelated to quality of bowel preparation were excluded: 17 had tumor obstruction, 3 had severe florid ulcerative colitis and 1 had a drug allergic reaction (Figure 1). The mean age of the study population was 60.1 ± 14.0 years, 256 (51.2%) were males and 305 (60.9%) received secondary education or higher. Majority of the cases were outpatients (70.6%) and performed in the morning (84.2%) (Table 1). The median colonoscopy appointment waiting time for cases was 15 weeks, with an interquartile range from 2 - 25 weeks. Over three-quarters of patients (76.4%) claimed that they were compliant to bowel preparation.

**Table 1:** Patient characteristics

Mean age, years	60.1 ± 14.0
**Men, n (%)**	256 (51.2)

**Education level, n (%)**	
**Primary or lower**	196 (39.1)
**Secondary or higher**	305 (60.9)

**In-patient or out-patient status, n (%)**	
**Out-patient**	354 (70.6)
**In-patient**	147 (29.4)

**Timing of colonoscopy, n (%)**	
**Morning**	421 (84.2)
**Afternoon**	80 (15.8)

**Colonoscopy appointment waiting time, n (%)**	
**< 1 week**	108 (21.6)
**2 - 15 weeks**	148 (29.5)
**> 16 weeks**	245 (48.9)

**Overweight or obese patient (based on BMI ≥ 23 for Asian population), n (%)**	266 (53.5)

**Presence of concomitant medical illness, n (%)**	222 (44.6)

**Patient with previous colonoscopy, n (%)**	161 (32.3)

**Patient with previous abdominal or pelvic surgery, n (%)**	134 (26.9)

**Compliant to bowel preparation, n (%)**	383 (76.4)

The indications for colonoscopy in patients included in this study were as follows: hematochezia (n = 92, 18.4%), colorectal carcinoma surveillance (n = 78, 15.6%), altered bowel habit (n = 73, 14.6%), colorectal carcinoma screening (n = 45, 9.0%), chronic constipation (n = 44, 8.8%), chronic diarrhea (n = 42, 8.4%), polyp surveillance (n = 38, 7.4%), suspected gastrointestinal malignancy (n = 34, 6.8%), abdominal pain (n = 20, 4.0%), obscure gastrointestinal bleeding (n = 20, 4.0%) and colitis assessment (n = 15, 3.0%). A total of 28 endoscopists performed the colonoscopies during the study period (10 senior endoscopists and 18 trainee endoscopists).

### Risk Factors Associated With Poor Bowel Preparation

The quality of bowel preparation identified in this study was as follows: good n = 45 (9%), intermediate n = 305 (60.9%) and poor n = 151 (30.1%). Among the 151 patients who had a poor bowel preparation, 71 (47%) failed to comply with bowel preparation instructions completely. Risk factors associated with poor bowel preparation were analyzed and indentified as follows: age 65 years old and above, lower education level (no formal education or primary education only), in-patient status, waiting time for colonoscopy appointment beyond 16 weeks and non-compliance to bowel preparation instructions (Table 2). Using logistic regression analysis, we identified lower education level, waiting time for colonoscopy appointment beyond 16 weeks and non-compliance to bowel preparation instructions as independent risk factors for poor bowel preparation (Table 2).

**Table 2:** Risk factors associated with poor bowel preparation on univariate and multivariate analyses

Patient characteristics	Quality of bowel preparation, n (%)		Unadjusted OR	95% CI	p value	Adjusted OR	95% CI	p value
							
	Poor n = 151	Non-poor n = 350						
**Age**								
**< 65 years**	80 (25.7)	231 (74.3)	1.00					
**≥ 65 years**	71 (37.4)	119 (62.6)	1.74	1.19, 2.56	0.005	1.36	0.87, 2.10	0.17

**Gender**								
**Female**	69 (21.2)	176 (71.8)	1.00			-	-	-
**Male**	82 (32.0)	174 (68.0)	1.20	0.82, 1.76	0.346			

**Education level**								
**≥ Secondary**	62 (20.5)	241 (79.5)	1.00			1.00		
**≤Primary**	89 (44.4)	109 (55.6)	3.10	2.09, 4.61	< 0.001	2.35	1.54,3.60	< 0.001

**Status**								
**Out-patient**	90 (25.4)	264 (74.6)	1.00			1.00		
**In-patient**	61 (41.5)	86 (58.5)	2.08	1.39, 3.12	< 0.001	1.09	0.66, 1.81	0.73

**Timing of colonoscopy**								
**Morning**	132 (31.4)	289 (68.6)	1.00			1.00		
**Evening**	19 (23.7)	61 (76.7)	0.68	0.39, 1.19	0.176	0.70	0.37, 1.32	0.70

**Appointment waiting time (weeks)**								
**< 1**	23 (21.3)	85 (78.7)	1.00			1.00		
**2 - 15**	47 (31.8)	101 (68.2)	1.72	0.97, 3.06	0.065	1.52	0.81, 2.87	0.19
**> 16**	81 (33.1)	164 (66.9)	1.83	1.07, 3.11	0.027	1.86	1.04, 3.37	0.035

**BMI**								
**≥ 23**	82 (30.8)	184 (69.2)	1.00			-	-	-
**< 23**	68 (29,4)	163 (70.6)	0.94	0.64, 1.38	0.736			

**Medical illness**								
**No**	73 (27.2)	195 (72.8)	1.00			1.00		
**Yes**	75 (32.6)	155 (67.4)	1.29	0.88, 1.90	0.192	1.05	0.67, 1.62	0.84

**Previous surgery**								
**No**	101 (28.0)	260 (72.0)	1.00			1.00		
**Yes**	49 (35.5)	89 (64.5)	1.42	0.93, 2.15	0.101	1.17	0.73, 1.89	0.51

**Previous colonoscopy**								
**No**	102 (30.2)	236 (69.8)	1.00			-	-	-
**Yes**	48 (29.8)	113 (70.2)	0.98	0.65, 1.48	0.934			

**Compliant to bowel preparation**								
**Yes**	80 (20.9)	303 (79.1)	1.00			1.00		
**No**	71 (60.2)	47 (39.8)	5.72	3.67, 8.91	< 0.0001	4.76	3.0, 7.55	< 0.001

### Impact of Poor Bowel Preparation on Technical Performance and Patient Comfort

**Figure 2:**
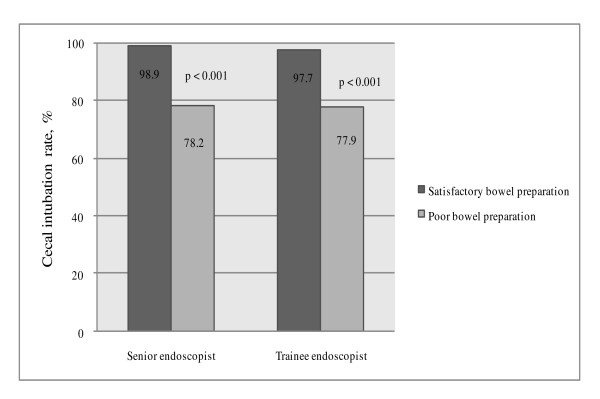
**Cecal intubation rate**. Cecal intubation rate was significantly lower in patients with poor bowel preparation regardless of the seniority of the endoscopist.

Poor bowel preparation was significantly associated with a decreased cecal intubation rate, prolonged cecal intubation time, prolonged total colonoscopy time and increased amount of flushing required regardless of whether the colonoscopy was performed by a senior or a trainee endoscopist (Figure 2 and Table 3). Poor bowel preparation was also associated with lower adenoma detection (13.2%) compared to non-poor bowel preparation (15.4%) although this was not statistically significant (p = 0.527).

**Table 3:** Cecal intubation time, total colonoscopy time and amount of flushing required

	Quality of bowel preparation		p value
		
	Satisfactory	Poor	
**Senior endoscopist**			

Cecal intubation time, min	11.12 ± 7.80	17.16 ± 9.41	< 0.001

Total colonoscopy time, min	18.78 ± 12.01	26.67 ± 12.05	< 0.001

Amount of flushing required, ml	84.05 ± 103.59	251.92 ± 121.66	< 0.001

**Trainee endoscopist**			

Cecal intubation time, min	22.35 ± 9.25	30.21 ± 11.78	< 0.001

Total colonoscopy time, min	33.75 ± 10.91	42.88 ± 15.48	< 0.001

Amount of flushing required, ml	137.13 ± 108.88	249.49 ± 131.23	< 0.001

Cecal intubation time, total colonoscopy time and amount of flushing required were inversely related to quality of bowel preparation regardless of the seniority of the endoscopist.

**Figure 3:**
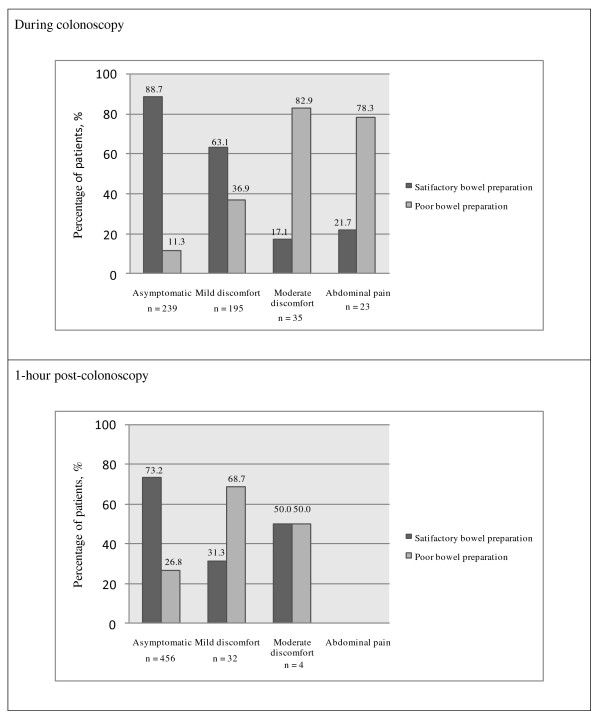
**Patient discomfort during and 1-hour post-colonoscopy**.

Poor bowel preparation was associated with increased patient discomfort during and up to one hour post-colonoscopy (Figure 3). 32.2% of patients with poor bowel preparation had moderate to severe abdominal discomfort during colonoscopy compared to only 3.2% of patients with good/intermediate bowel preparation (p < 0.001). 16.4% of patients with poor bowel preparation had mild to moderate abdominal discomfort one hour post-colonoscopy compared to only 3.4% of patients with good/intermediate bowel preparation (p < 0.001). There was no significant difference in the amount of sedation received by the patients with the different grades of bowel preparation. The association of increased patient discomfort with poor bowel preparation during and 1-hour post-colonoscopy was independent of the indication for the procedure and the total colonoscopy time (data not shown).

## Discussion

Quality issues pertaining to colonoscopy remain important in the clinical application of this modality. In Asia, particularly in less developed countries, healthcare resources remain limited and inappropriate utilization of these resources can have serious implications for vast numbers of the population. As poor bowel preparation clearly has a significant impact on the utility of colonoscopy, data from our study provides useful information both in Asia and in other resource-limited healthcare systems.

The patients enrolled in this study were derived from both primary and secondary care. As our endoscopy unit operates an open-access policy, approximately 40% of referrals from colonoscopy are from the primary care clinics attached to this institution [15]. Hence data from this study is generalizable to populations scheduled for colonoscopy at large. Over one-third of the patients had no formal education or only received primary education. This is not unexpected as the study population consisted of a significant proportion of elderly patients who had limited educational opportunities during the nation's economic development in the early decades of the twentieth century [16].

Almost one third (30.1%) of patients undergoing colonoscopy in our centre had poor bowel preparation despite using a standard, well-tested bowel preparation method. This proportion of patients with poor bowel preparation appeared comparable to studies that have been conducted in developed countries. In a retrospective study of a US endoscopy database, Harewood et al reported 23.1% inadequate bowel preparation amongst adults undergoing colonoscopy in various centres [5]. In another retrospective study of 12,430 cases from the US, the proportion of suboptimal bowel preparation was found to be as high as 34% in patients on Medicaid [17]. Poor preparation was found to be present in 28.2% among 362 patients compliant to bowel preparation instructions in another Korean study [10].

Less than half (47%) of patients who had poor bowel preparation failed to comply with bowel preparation instructions in this study. This suggests that other factors additionally contributed towards poor bowel preparation in this study. Indeed, although failure to comply with preparation instructions was identified as one of the more significant predictors for poor bowel preparation (OR 4.73) other factors among our study population were found to be independent predictors for a poor bowel preparation. Adults with lower education levels were twice as likely to have a poorer bowel preparation compared to patients without these factors. Adults with lower education levels may have had a lack of understanding of the importance of adhering to bowel preparation instructions and it is clear that our healthcare system has to find a mechanism to compensate for this problem. However, this problem may not be unique to populations in developing countries alone. In a recent retrospective survey of 12,430 US patients who had undergone colonoscopy over a 28 month period, Lebwohl and colleagues demonstrated that patients on Medicaid, i.e. with a lower socioeconomic status, had a significantly higher level of suboptimal bowel preparation compared to patients not on Medicaid [17]. Being on Medicaid alone was identified as an independent predictor of suboptimal bowel preparation in this study.

To our knowledge, a prolonged appointment waiting time has never been reported as a predictor of poor bowel preparation before. We observed that patients with a prolonged appointment time, longer than 16 weeks in this study, were twice as likely to have a poor bowel preparation. With the increasing demands on colonoscopy services and finite resources world-wide, our findings have a universal implication. It is very probable that details of bowel preparation instructions can be forgotten with the passage of time, despite the reminders of written instructions or brochures. The latter may get misplaced over time or the details get misinterpreted. Greater resources i.e. endoscopy staffing and more equipment and lists, is one way to minimize waiting times. Additionally, employing more support staff to contact patients and remind them about bowel preparation instructions are possible solutions to this problem.

The impact of poor bowel preparation on colonoscopy performance and patients' level of discomfort are similar to previously reported [6]. Regardless of endoscopists' experience, we demonstrated that poor bowel preparation resulted in a reduced cecal intubation rate and prolongation of colonoscopy time. Poor bowel preparation has been associated with reduction in diagnostic yield [4-6,18-20]. Although our study did not show a significant difference in adenoma detection between poor and non-poor bowel preparations, we did find a trend towards lower adenoma detection among patients with poor bowel preparation. Furthermore, poor bowel preparation additionally resulted in more patient discomfort as sedation doses were similar in patients with and without poor preparation. Increased levels of discomfort experienced by the patient may impede willingness to undergo repeat examinations when indicated and reduce acceptability of colonoscopy in the public generally.

This study had several limitations. We did not use a validated bowel grading scale, such as those that have been developed in Otawa [21] and Boston [22]. However, the terminology that was used for reporting, i.e. excellent, good, fair and poor, is still deemed acceptable by a recent American Society for Gastrointestinal Endoscopy (ASGE) and American College of Gastroenterology (ACG) Taskforce on Quality in Endoscopy [8]. Furthermore, we addressed the issue of inter-observer variability, with a re-categorization into a simplified scale (i.e. good, intermediate and poor) for the purposes of analysis. Reliance on patients or their relatives for information on compliance to bowel preparation instructions was another limitation. Nevertheless, we have conducted a large study and the magnitude of the effect of compliance on risk of poor bowel preparation is unlikely to be completely explained by bias or confounding factors. In assessment of cecal intubation time and total colonoscopy time, we did not make adjustment for time spent to perform therapeutic work. As the total number of patients with lesions requiring biopsies were small in this cohort of patients (23.4%), we believe that not adjusting for procedures would not make a significant difference on the overall measurement of colonoscopy performance. The assessment of patient discomfort, which was based on patient recollection, could potentially have been affected by anterograde amnesia following Midazolam administration. This effect may have influenced the perception of discomfort perceived by patients during colonoscopy and to a lesser extent for 1-hour post-procedure. Despite this limitation, we still observed a significant difference in level of discomfort between patients with and without poor bowel preparation.

## Conclusions

In conclusion, we identified that 30.1% of our multi-racial Asian population had poor bowel preparation at the time of colonoscopy. Whilst non-adherence to bowel preparation instructions was a major factor, this study indentified that low education levels and prolonged appointment waiting times were independent predictors of poor bowel preparation. Poor bowel preparation adversely affected technical performance of colonoscopy and resulted in greater patient discomfort. At the institutional level, greater effort is required to address prolonged waiting times and Asian patients with lower education levels. These measures are mandatory to improve the efficacy of colonoscopy as a diagnostic and therapeutic tool in colonic disease in this population.

## Competing interests

The authors declare that they have no competing interests.

## Authors' contributions

WKC interpreted the data and drafted the manuscript. AS designed the study, collected data and interpreted the data. JM contributed to data collection. KLG provided administrative support. SM contributed to study design and data interpretation and did the final review of the manuscript. All authors have read and approved the final manuscript.

## Pre-publication history

The pre-publication history for this paper can be accessed here:

http://www.biomedcentral.com/1471-230X/11/86/prepub
